# High throughput AS LNA qPCR method for the detection of a specific mutation in poliovirus vaccine strains

**DOI:** 10.1016/j.vaccine.2024.01.103

**Published:** 2024-04-02

**Authors:** Lizet Opmeer, Isabella Gazzoli, Mónika Ballmann, Marieke Willemsen, Gerben P. Voshol, Magda Grudniewska-Lawton, Menzo Havenga, Christopher Yallop, Ahd Hamidi, Gert Gillissen, Wilfried A.M. Bakker

**Affiliations:** a Batavia Biosciences B.V., Bioscience Park Leiden, Zernikedreef 16, 2333CL Leiden, The Netherlands; b GenomeScan B.V., Plesmanlaan 1d, 2333 BZ Leiden, The Netherlands

**Keywords:** Quality control, sIPV, Sabin Poliovirus 2, qPCR, Vaccine, AS LNA qPCR, Mutations, NGS, VP1-E295K

## Abstract

Sabin Inactivated Poliovirus Vaccine (sIPV) has become one of the preferred vaccination options for the last step in the Poliovirus eradication program. Sequencing of poliovirus samples is needed during the manufacturing of poliovirus vaccines to assure the safety and immunogenicity of these vaccines. Next-generation sequencing analysis is the current costly and time-consuming gold standard for monitoring the manufacturing processes. We developed a low-cost and quick, highly sensitive, and allele-specific locked nucleic acid-probe-based reverse transcription quantitative PCR alternative that can accurately detect mutations in poliovirus vaccine samples during process development, scaling up, and release. Using the frequently *in vitro* occurring and viral replication-impacting VP1-E_295_K mutation as a showcase, we show that this technology can accurately detect E_295_K mutations in poliovirus 2 samples to similar levels as NGS. The qPCR technology was developed employing a synthetic dsDNA fragment-based standard curve containing mixes of E_295_K-WT (wildtype) and Mut (mutant) synthetic dsDNA fragments ranging from 1 × 10^7^ copies/µL to 1 × 10^2^ copies/µL to achieve a linear correlation with R^2^ > 0.999, and PCR efficiencies of 95–105 %. Individual standard concentration levels achieved accuracies of ≥92 % (average 96 %) and precisions of ≤17 % (average 3.3 %) RSD. Specificity of locked nucleic acid (LNA)-probes was confirmed in the presence and absence of co-mutations in the probe-binding region. Application of the developed assay to Sabin Poliovirus type 2 production run samples, illustrated a linear relationship with an R^2^ of 0.994, and an average accuracy of 97.2 % of the variant (allele)-specific AS LNA qPCR result, compared to NGS. The assay showed good sensitivity for poliovirus samples, containing E_295_K mutation levels between 0 % and 95 % (quantification range).

In conclusion, the developed AS LNA qPCR presents a valuable low-cost, and fast tool, suitable for the process development and quality control of polio vaccines.

## Highlights

• AS LNA qPCR can accurately identify VP1-E_295_K containing samples in sPV2 batches.
• The developed AS LNA qPCR for E_295_K mutation is highly sensitive.
• LNA probes used are highly specific in targeting known mutations.
• AS LNA qPCR is likely applicable to other vaccine strains, serotypes, and mutations.

## Nomenclature

AS: allele-specific
bOPV: bivalent oral poliovirus vaccines
BRAF: v-raf murine sarcoma viral oncogene homolog B1
cVDPV: circulating vaccine-derived polioviruses
LNA: locked nucleic acids
KRAS: Kirsten rat sarcoma virus
Mut: mutant
nOPV2: novel OPV2
PV: poliovirus
PV2: Poliovirus 2
RSD: Relative standard deviation
RT qPCR: reverse transcription quantitative real-time PCR
SD: Standard deviation
sIPV: Sabin strain inactivated Poliovirus vaccine
sPV2: Sabin Poliovirus 2
tOPV: trivalent live-attenuated oral poliovirus vaccines
VP: capsid V protein
wIPV: conventional inactivated poliovirus vaccine from wildtype strains
WT: wildtype

## 1 Introduction

Trivalent live-attenuated oral poliovirus vaccines (OPV), containing the three serotypes of poliovirus (Sabin 1–3) have led to the successful eradication of wild poliovirus types 2 and 3 in 2015 and 2019 [1], respectively, while wild-type-1 poliovirus (PV) remains endemic in Afghanistan and Pakistan [2], with its prevalence increasing in 2019 and 2020 [3]. However, recipients of the trivalent OPV can excrete revertant circulating vaccine-derived polioviruses (cVDPV) [4], [5], which cause most current polio outbreaks, particularly in poorly immunized communities (weekly updates per country: https://www.polioeradication.org/polio-today/polio-now/this-week/). In 90 % of the cases, these vaccine-derived circulating polioviruses are derived from the Sabin PV2 vaccine strain (cVDPV2). A switch to bivalent OPV (bOPV) in 2016 did not halt these cVDPV2-outbreaks. bOPV contains only serotypes 1 and 3, supplemented with at least one dose of conventional inactivated poliovirus vaccine from wildtype strains (wIPV), as wIPV protects from polio-induced paralysis. Yet, individuals receiving bOPV with wIPV can still be infected with and transmit type 2 poliovirus and cVDPV [6], [7], [8]. In 2022, 675 cases of cVDPV2 were confirmed, an incidence that declined to 50 in 2023 (until 17th May) due to the recent development of improved versions of the IPV and OPV vaccines [9].

The immunogenicity and neurovirulence of OPVs are changed by mutations occurring in the internal ribosome entry site (IRES), which is located in the mRNA 5′ untranslated region (UTR), and the capsid region (composed of four capsid proteins, VP1-4) during the production of poliovirus vaccines. To circumvent these mutational problems, two novel OPV2 (nOPV2) vaccine candidates have recently been developed, one with a genetically stabilized IRES domain V [10] (candidate 1) and one with a genetically stabilized IRES domain V and an additional codon deoptimized capsid region [11] (candidate 2). This stability was achieved by the replacement of the weak U-G and selected strong C-G nucleotides with intermediately strong U-A pairs [12]. Yet, mutations during the manufacturing process can still occur, and lot release monitoring is required to prevent the release of low immunogenicity batches and chances for reversion to neurovirulence of nOPV2 vaccine candidates. Given the urgent public health need to address cVDPV2 in polio-affected countries, the nOPV2 vaccine has been made available through WHO’s Emergency Use Listing (EUL) procedure since 2020 [13].

In contrast, wild-type poliovirus strains used in conventional IPVs by definition are neurovirulent before inactivation, and therefore present a potential biosafety hazard should they escape during the manufacturing process. Also, their use is limited by the cost of production and insufficient supply for global needs [14]. When using attenuated strains as an alternative in IPV manufacturing, this biosafety hazard was considered significantly lower when compared with using wild-type stains. However, attenuated Sabin poliovirus strains may revert to neurovirulence before inactivation in rare cases (5′UTR nucleotide 481-G as key neurovirulence marker in Sabin PV2 [sPV2]) [15] and therefore still present a potential biosafety hazard [16] should they escape during the manufacturing process. Current manufacturing processes that use attenuated Sabin strains during vaccine production (sIPV) allow for process modernization and optimization, thereby increasing containment safety and reducing costs [17]. However, also these sIPVs need to be closely monitored for the occurrence of mutations. In *in-vitro* environments, the capsid region VP1-E_295_K mutation tends to accumulate in poliovirus type 2 (both sPV2 and nOPV2s) and was thus selected as a showcase for our variant (allele)-specific locked nucleic acid-based reverse transcription quantitative real-time PCR (AS LNA qPCR) technology. This E_295_K mutation is caused by a single nucleotide variant from guanine (G) to adenine (A), resulting in the codon change from GAA to AAA (translated to Glutamic Acid = E or Lysine = K, respectively). While glutamic acid has an acidic side chain, the side chain of lysine is basic at physiological pH. Seeing that for the Lansing PV2 strain, VP1-295 is located in the CD155 receptor-binding domain [18], it is hypothesized that this difference in charge can lead to a conformational change of the VP1 capsid protein, causing loss of replicative fitness as shown in Hep-2c and Vero cells, and in murine experiments [16]. In turn, this loss of replicative fitness through the VP1-E_295_K mutation negatively impacts the sIPV manufacturing process and this mutation thus needs to be closely monitored during the manufacturing process.

In the past few years, next-generation sequencing (NGS) technology is being used to monitor the vaccine manufacturing process and detect mutations in Polio vaccines, including sIPV [19]. This method can identify even low-rate mutations and mutations not detectable with classical mutational analysis methods, including MAPREC [20], [21], but is also associated with great costs from workflow (hands-on time) and laborious data analysis. To maintain cost-effective manufacturing processes, we here present a proof-of-principle of a targeted, allele-specific quantitative PCR that can be employed for pre-screening of allelic variants before and even instead of costly NGS. Of note, allele-specific quantitative PCR has been successfully demonstrated to detect VP1-I_143_T, VP1-N_171_D, and VP1-E_295_K mutations at comparable accuracy to NGS in the nOPV2 setting [22].

In this work, we developed an AS LNA qPCR method to specifically detect and quantify the most prominently occurring mutation in sPV2 during sIPV manufacturing. We show proof of the technology for the E_295_K mutation in attenuated sPV2 in Poliovirus bulk during sIPV vaccine production. Our results demonstrate the significance of using oligo probe LNA (locked nucleic acid qPCR probe) for accurate and highly specific detection of mutations, as pre-screening and potentially replacement analysis to the NGS for screening genetic variants in in-process Poliovirus samples.

## 2 Materials and methods

### 2.1 Viruses

For the production of sPV2 batches, a plasmid-derived viral seed was utilized, which was generated by Batavia Biosciences through a combination of viral passaging and clone selection. The plasmid itself was synthesized by Gene Universal, located in Delaware, USA, and designed to match the WHO international reference material sequence for poliovirus Sabin type 2 (NIBSC code: 01/530, GenBank AY184220).

The sPV2 batches used in this study were available from in-house resources, derived from development and standardized production runs between 2020-today. The batches were stored as frozen clarified or purified and formulated viral samples at −80 °C until use. Clarification of the viral samples was conducted by centrifugation at 600–1000× *g* for at least 5 min, to pellet the cells. The matrix for these samples was either the serum-free M199-based or the serum-free DMEM-based Batavia Biosciences’ infection media, used for the virus production phase. For purified and formulated batches, the matrix was M199 supplemented with 5 g/L glycine. Experimental and control samples were selected based on the presence and absence of E_295_K, respectively, as originally assessed by NGS. sPV2 batches were thawed before the subsequent processing.

### 2.2 Viral RNA isolation

Viral RNA isolation was performed using the MagMAX™ Viral RNA Isolation Kit (Thermo Fisher Scientific, Applied Biosystems™, Cat# AM1939), for all purposes (AS LNA qPCR and NGS), mostly according to the manufacturer’s instructions. Exceptions were: For all isolations, 1.6 µL linear acrylamide (Thermo Fisher Scientific, Invitrogen™, Cat# AM9520) was used as a coprecipitant per sample instead of the 2 µL Carrier RNA provided by the MagMAX kit because the latter may interfere with the NGS reaction. Upon isolation of the RNA, the RNA concentration was determined using a NanoDrop 2000 spectrophotometer (Thermo Fisher Scientific, Thermo Scientific™) for RT-PCR purposes. For NGS, RNA concentration was measured based on fluorescence using the Qubit RNA HS Assay kit (Thermo Fisher Scientific, Invitrogen™ Cat# Q32852) and the Qubit 3.0 Fluorometer (Thermo Fisher Scientific, Invitrogen™, Cat# Q33216).

### 2.3 Primers and LNA probes

All primers, probes, and synthetic dsDNA fragments (gBlock Gene Fragments, IDT) were purchased from IDT – Integrated DNA Technologies (Coralville, Iowa, United States). The sequence used to design the primers, probes, and gBlocks around the E_295_K mutation is shown below (positions 3271–3450 of GenBank AY184220). The G/A mutation is marked in red, additional SNPs found in our dataset within the target sequence are presented in green and blue, and primer and probe sequences are underlined. The resulting amplicon size is 97 bp, covering positions 3303–3399 of the reference sequence:

**Figure:**
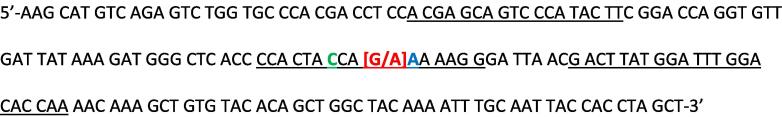

Primer and probe sequences were evaluated using the OligoAnalyzer tool provided by IDT. The analysis indicated no significant secondary structure interactions between the primers and probes. Additionally, when the sequences were subjected to BLAST analysis against the target species (Human poliovirus), no off-target amplification sequences were predicted. LNA probes were purchased as HPLC-purified at the following scales as FAM-E_295_K-Mut: 250 nmole Affinity Plus 5′G-FAM™3′IBFQ (16 bases) and HEX-E_295_K-WT: 250 nmole Affinity Plus 5′G-HEX™3′IBFQ (14 bases). Both E_295_K-WT (wildtype) and Mut (mutant) probes contained 6 Affinity Plus bases (LNAs; locked nucleic acids), which have an increased affinity for their complementary strand, leading to increased hybridization melt temperature (Tm). Consequently, these LNA probes can be designed with shorter lengths than standard qPCR probes. Shorter probes have better quenching and a higher signal-to-noise ratio and thus have an improved ability to distinguish SNPs. The LNA bases are indicated with a “+” in Table 1. The WT probe contained an LNA G base at the position of interest (indicated in red), while the Mut probe contained an LNA A base at the position of interest (red). The WT probe was labeled at the 5′-end with a HEX fluorescent dye, and the Mut probe was labeled at the 5′-end with a FAM fluorescent dye. Both probes were quenched with a 3′ Iowa Black® Fluorescent Quencher.

**Table 1:** Primers, probes, and synthetic dsDNA fragments (gBlocks, IDT) used. In red: location responsible for the E_295_K mutation. G is the wild-type allele. In blue: location of co-mutation “E_295_G”, A → G. In green: location of co-mutation “P_294_S”, C → T. WT – wild-type, Mut – mutant, FOR – forward, REV – reverse. Preceding ‘+’ indicates the location of LNA-bases. The gBlock for SNPs E_295_G and P_294_S were synthesized while preserving the E_295_ in WT, because co-mutation analysis revealed that the E_295_K mutation did not co-occur with one of the other mutations on a single viral genome.

### 2.4 AS LNA qPCR

The AS LNA qPCR was performed as two independent reactions; a random reverse transcription step and an allele-specific qPCR step.

#### 2.4.1 Reverse transcription

The input quantity for each reaction was 20 ng of viral RNA. DNAse treatment, annealing of random primers, and reverse transcription were performed using the SuperScript™ IV First-Strand cDNA Synthesis System with ezDNase Enzyme kit (Invitrogen, Cat# 18091150). All mixes were prepared according to the manufacturer’s instructions, with the following specifications: DNase incubation was allowed for 2 min at 37 °C followed by 2 min on ice. Random hexamer annealing was performed at 65 °C for 5 min. Reverse transcription was completed after cooling the samples for at least 2 min on ice (Anneal: 23 °C, 10 min; cDNA synthesis: 50 °C, 10 min; RT enzyme inactivation: 80 °C, 10 min; Hold: 4 °C, up to 60 min) using a thermal cycler (Biometra TRIO 48, Analytik Jena).

#### 2.4.2 Allele-specific qPCR

Unless otherwise specified, cDNA was standardly diluted 10-fold in nuclease-free distilled water (Gibco, 10977-035), of which 5 µL was used as input per qPCR reaction. The qPCR was performed using the PrimeTime Gene Expression Master Mix from IDT (Cat# 1055772). The appropriate amount of reference dye was added to the master mix, matching the qPCR system used, as described in the product manual. The qPCR reaction master mix was prepared as per the following final concentrations: PrimeTime Gene Expression master mix (2X) = 1X; primers (E_295_K-FOR and E_295_K-REV) = 0.50 µM each; HEX-E_295_K-WT probe = 0.25 µM**;** FAM-E_295_K-Mut probe = 0.20 µM. After dispensing 15 µL master mix per well in 96-well MicroAmp™ Fast or non-Fast Optical 96-Well Reaction Plate (Applied Biosystems, Cat# 4346906), 5 µL sample, standard or control was added, and the plate was sealed with an optically transparent film (MicroAmp™ Optical Adhesive Film, Applied Biosystems, Cat# 4311971). Each standard, control, or sample condition was tested in triplicate wells to help ensure the reproducibility and reliability of the results. The plate was centrifuged briefly (600–1000× *g* for 1 min) before running the cycling protocol in the Applied Biosystems™ QuantStudio5 Real-Time PCR System (3 min at 95 °C [polymerase activation], then 40 cycles of 15 sec at 95 °C [denaturation], 1 min at 63 °C [annealing/extension: tested conditions were 59 °C–68 °C using the VeriFlex function with Delta 1 °C]). All wells of the qPCR plates were set to detect both HEX and FAM dyes in the QuantStudio™ Design & Analysis Software v1.5.2 (for the HEX-E_295_K-WT and FAM-E_295_K-Mut probes, respectively). For detection of the HEX-E_295_K-WT probe, a custom dye calibration was performed using a HEX-labeled oligo dT, according to the QuantStudio5 manual (oligo(dT)10: 5′-HEX/TTTTTTTTTT-3′, IDT, LabReady and HPLC purified). For both probes non-fluorescent quencher (NGF) was selected as a quencher in the software, as advised by IDT for the Iowa Black FQ. ROX was set as a passive reference dye for all wells, for background correction.

#### 2.4.3 Synthetic dsDNA fragments for standard curves and controls

The gBlocks were synthesized by IDT (250 ng each) and resuspended according to the manufacturer’s instructions, to reach a final concentration of 10 ng/µL (stock solutions). Two 10 µL aliquots of the stock solutions of all gBlocks were stored at −20 °C. All gBlocks (Table 1) were resuspended and diluted in TE buffer pH 8.0 + 0.5 mg/mL Poly(A) to 1 × 10^9^ copies/µL working stocks, which were stored at −20 °C until use. To convert from nanograms to copy numbers, the molecular weight (as provided in Table 1), the Avogadro constant (6.022 × 10^23^), and Equation S1 (refer to supplementary equations file) were utilized.

The gBlock working stocks were used for preparing qPCR standard curves and control samples. Standard curve ranges were fixed to serial 10-fold dilutions ranging from 1 × 10^7^ copies/µL to 1 × 10^2^ copies/µL of both the WT and the Mut gBlocks. Except for optimization experiments, mixes of WT and Mut gBlock standard curves were tested (in other words WT and Mut were mixed in the same well). E_295_K-WT and -Mut gBlocks were also used as sources for the control samples at different mutation percentages (Mut%), ranging from 0 % E_295_K to 100 % E_295_K. The total input of E_295_K-WT plus Mut gBlock per qPCR reaction varied from 1 × 10^3^ copies/µL to 1 × 10^5^ copies/µL. Unless otherwise specified, the total input was fixed at 1 × 10^5^ copies/µL and the diluent for preparing the standard curve or control dilutions was nuclease-free distilled water. In case ready-to-use control samples were prepared in advance, the diluent was TE buffer pH 8.0 + 0.5 mg/mL Poly(A), and aliquots were stored at −20 °C until use. E_295_G and P_294_S gBlocks were also synthesized by IDT, resuspended, and diluted similarly to E_295_K-WT and -Mut gBlocks.

### 2.5 Data analysis

The thresholds and baselines for the analysis of both probes were set automatically in the QuantStudio software. Before setting the thresholds and baselines, any empty wells were omitted. All results were exported from the QuantStudio software to Microsoft 365 Excel (Microsoft Corporation). The WT and Mut standard curves were separately analyzed in Excel, by using the SLOPE and the INTERCEPT functions for a linear regression line with Log quantities on the X-axis and Ct values on the Y-axis. The slope and intercept values (also calculated in the QuantStudio software) were then used to perform back-calculations of the individual standard point quantities (see Equation S2 of the supplementary equations file). Next, the back-calculated quantities (average of triplicate measurements) were used to calculate the accuracy and precision of the standard points (see Equations (1), (2), (3)). The coefficients of determination (R^2^) and the PCR efficiencies (also refer to Equation S3 of the supplementary equations file) of the standard curves were computed using the QuantStudio software.

Equation (1): Deviation of standard points

(1) $$\mathrm{Deviation}\;\left[\%\right]=\left|\frac{\mathrm{Average}\;quantity\;result\;\left[copies/\mu L\right]\;}{\mathrm{Quantity}\;input\;\left[copies/\mu L\right]}-1\;\right|\times 100\%$$

Equation (2): Accuracy of standard points, controls or samples

(2) $$\mathrm{Accuracy}\;\left[\%\right]=100\%-Deviation\;\left[\%\right]$$

Equation (3): Precision of standard points (quantity) or gBlock control samples (Mut%) (SD = standard deviation)

(3) $$\mathrm{Precision}\;\left[RSD\right]=\frac{\mathrm{SD}\;of\;triplicate\;measurements}{\mathrm{Average}\;of\;triplicate\;measurements}\times 100\%$$

The poliovirus samples and gBlock control samples were analyzed using the quantities, as calculated by the qPCR software, based on the WT and Mut standard curves. The E_295_K mutation percentage (Mut%) was calculated according to Equation (4). The variation of the E_295_K Mut% was monitored by applying a specification of a maximum 5 % E_295_K Mut% difference over the triplicate measurements (Equation (5)). Thereafter the accuracy of the E_295_K RT-qPCR result was calculated by comparing it to the NGS results, resulting in the Mut% difference (Equation (6)). In some cases, also the absolute Mut% difference was calculated, using the ABS function in Excel, for example for the calculation of the absolute Mut% difference between two runs.

Equation (4): E_295_K mutation percentage (Mut%)

(4) $$\mathrm{Mut}\%=\frac{\mathrm{Mut}\;quantity}{\mathrm{Mut}\;quantity+WT\;quantity}\times 100\%$$

Equation (5): Variation of RT-qPCR result

(5) %Mutvariationoftriplicatemeasurements=MaximumMut%-MinimumMut%

Equation (6): Deviation of RT-qPCR result

(6) $${\mathrm{Mut}\%\;difference}^{*}\;or\;Deviation=qPCR\;result\;-Mut\%\;input\;or\;NGS\;results$$

* Difference of RT-qPCR Mut%, compared to input (for gBlock control samples) or NGS results (for sPV2 samples).

### 2.6 NGS and sequencing analysis

Short read NGS was outsourced to GenomeScan B.V. Briefly, sample preparation was performed using the NEBNext Ultra II Directional RNA Library Prep Kit for Illumina (NEB #E7760S/L) following the manufacturer's protocol. The cDNA synthesis was followed by ligation with sequencing adapters and PCR amplification of the resulting product. The quality and yield of the prepared samples were measured using the Fragment Analyzer (Agilent). The size distribution of resulting products was consistent within the expected range of 300–500 bp. DNA sequencing and clustering were performed using the NovaSeq6000 (Illumina) according to the manufacturer's protocols, with a DNA concentration of 1.1 nM. The NovaSeq control software NCS v1.8 was used.

#### 2.6.1 NGS data analysis

Primary data analysis, including image analysis, base calling, and quality check, was performed using the Illumina data analysis pipeline RTA3.4.4 and BclConvert v3.10.5 (see supplementary for raw data tables, including amplicon sequence data). To mitigate the effects of base calling errors, bases from FASTQ reads with phred scores below Q30 were trimmed off. Any resulting reads shorter than 36 bp were excluded. The reads were subsequently mapped using BWA v0.7.4 to the strain-specific reference, and the output files consisted of BAM files to represent aligned sequences. Subsequently, TSV files showing variant frequencies mapped against the reference sequence in a tabular format were generated using a customized in-house version of PaCBAM (based on v1.6.0). Sample data quality was assessed by the coverage (number of counts/position). An average coverage of ≥1000 was considered to reflect sufficient data quality. All positions, showing coverage below 1000 counts, were interpreted cautiously. Below 100 counts, the sample data was considered invalid. For each variant of every position, the strand value was calculated. Extreme strand bias indicates a potentially high chance for false-positive variant detection [23]. The strand value indicates if the variant was found equally in forward and reverse reads (strand value = 0.5) or if the variant was primarily found at either forward (strand value > 0.5) or reverse reads (strand value < 0.5). So, a strand value of 1 means that 100 % of the reads were generated in the forward direction, while a strand value of 0 means that 100 % of the reads were generated in the reverse direction. Around the E_295_K mutation, it was observed that the strand value is generally around 0.7, indicating that the majority of the reads were generated in the forward direction. This bias towards forward direction read generation was not considered an issue, since this area of the poliovirus genome has been studied extensively, using different techniques, allowing for confidence in the mutation data obtained.

## 3 Results

### 3.1 Probe-based AS LNA qPCR assay design

The presented AS LNA qPCR for the detection of E_295_K was developed as two independent reactions; the first step is the reverse transcription reaction, which produces a non-specific cDNA population from the RNA sample, using reverse transcriptase and random primers. The second step is a qPCR, that specifically amplifies the targeted cDNA using forward and reverse primers and HEX- (E_295_K-WT) or FAM- (E_295_K-Mut) labeled LNA-probes, which show improved mismatch discrimination compared to traditional qPCR probes. In the qPCR step, amplification only occurs when the primer-probe set recognizes its specific target and generates a signal, while no amplification occurs with a non-specific target. The designed qPCR probes are specific for the wild type (E) or the mutant (K) versions of the E_295_K SNP in the VP1 gene of poliovirus type 2, allowing each for detection of the targeted variant (WT or Mut) in a single and multiplex qPCR (HEX and FAM dyes). ROX was included as a passive reference dye in each reaction, for normalization of the fluorescence levels.

### 3.2 Robustness of the probe-based qPCR design

For the qPCR runs, performed according to the final assay protocol, the following acceptance criteria were used to evaluate the validity of the data. 1) Standard curve R^2^ ≥ 0.995 and the PCR efficiency should be 90–110 %. 2) Controls should have an absolute mutation percentage (Mut%) difference, compared to input, of <10 % (Equation (6)). 3) Samples should have a maximum difference of 5 % E_295_K Mut% between the maximum and minimum E_295_K frequencies (triplicate measurements). Sample WT and/or Mut quantity (average of triplicate measurements) should fall within the WT and Mut standard curve ranges, respectively. 4) Average Ct of the no template control (negative control) should be undetermined, or “Ct negative control - average Ct Std 6” ≥1.0 Ct. For all the assay runs in this paper, performed according to the final protocol, the predefined acceptance criteria were reached, concluding that the assay provides consistent data quality and that the obtained results could be used to predict the general assay performance (i.e., precision, linearity, accuracy).

**Fig. 1:**
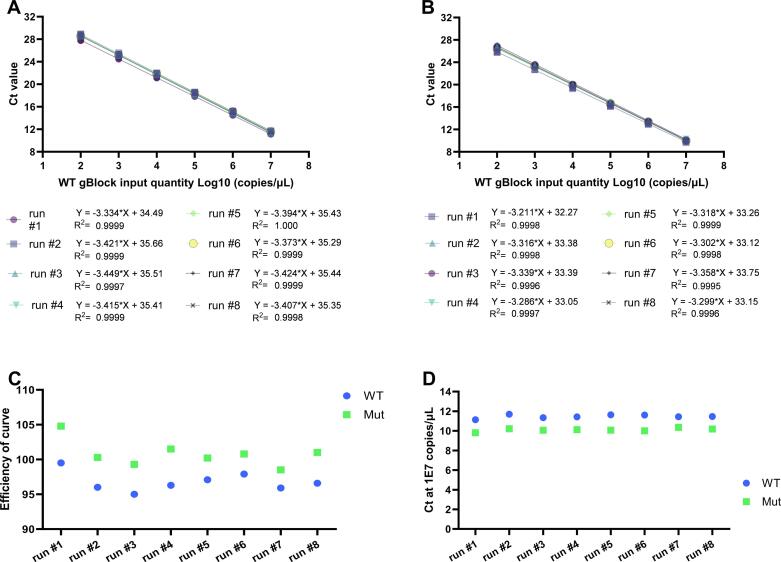
Amplification efficiency, linearity, and reproducibility of the developed probe-based qPCR. Linearity and R^2^, as a coefficient of the correlation, of the (A) WT and (B) Mut standard curves with measured outcomes in 8 separate runs. (C) The efficiency of the qPCR reactions of WT and Mut standard curves in 8 separate runs. Average WT and Mut amplification efficiencies are presented in the right two graph points including SD. (D) Ct value at 1 × 10^7^ copies/µL Wt or Mut gBlocks in 8 separate runs. Average WT and Mut amplification efficiencies are presented in the right two graph points including SD. For all graphs: Negative controls were not detectable in all runs; data not shown.

As depicted in Fig. 1A, B, and C, the AS LNA qPCR efficiencies ranged from 95 to 105 %, with an average R^2^ of >0.999, and passed the initial acceptance criteria in 8 separate runs. Ct comparison between the different runs at 1 × 10^7^ gBlock copies/µL standard points, visualized in Fig. 1D, revealed that on average the E_295_K-WT gBlock resulted in a Ct of 11.5 and the E_295_K-Mut gBlock Ct was on average 10.1. Initially, the E_295_K-WT and the E_295_K-Mut standard curves were tested separately, but efficiencies and R^2^ as analyzed for the mixed standard curves were comparable to those with the separate standard curves (data not shown). Therefore, we proceeded with mixed standard curves for all runs shown in this correspondence. Importantly, the mixed standard curve contained mixes of the E_295_K-WT and -Mut gBlocks in a 1:1 ratio. This way the probe competition occurring in the samples is similar to the standard wells.

### 3.3 High linearity, precision, and specificity for detecting E_295_K mutations

**Fig. 2:**
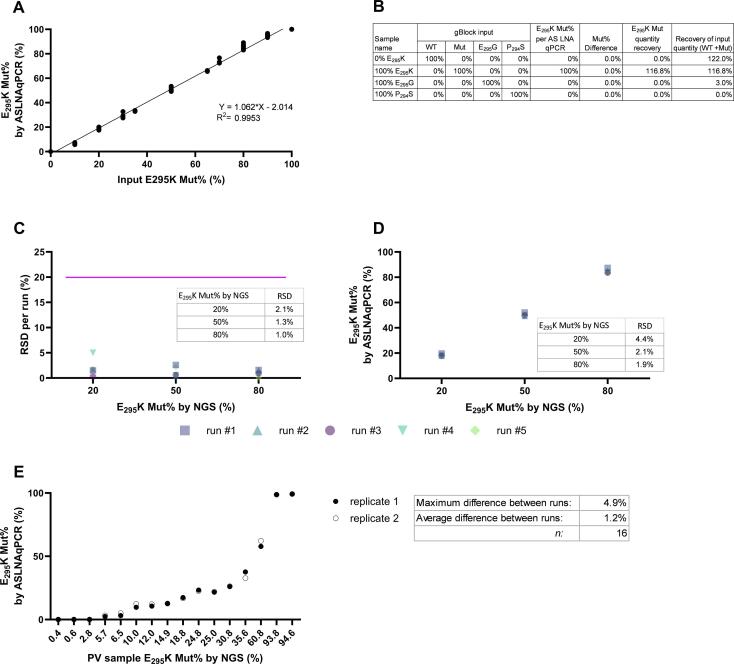
High linearity, precision, and specificity for detecting E_295_K mutations. (A) Correlation of input E_295_K mutation frequency (Mut%) with measured E_295_K mutation frequency. (B) Mut% recovery in cases of co-mutation standard curves. (C) Variation within 1 run expressed as RSD (precision) per input E_295_K%. (D) Variation between runs (*n* = 5). 3 technical replicates were averaged resulting in one data point per run. (E) Difference between runs (*n* = 2, 16 sPV2 samples) with increasing levels of E_295_K Mut%. All experiments were performed using the Applied Biosystems™ QuantStudio5.

Next, we tested the linearity and precision of the assay with synthetic gBlock samples, mixed at different ratios, to aim for E_295_K Mut% varying from 0 % to 100 % E_295_K Mut%. To test for precision we selected low, middle, and high levels at 20 %, 50 %, and 80 % E_295_K Mut%, respectively. Each level was tested 3 times independently across 5 runs. For linearity, we tested a range of 0 % to 100 % E_295_K Mut% with increments of 10–20 % between the levels. Three runs were performed, each testing 9 different levels of E_295_K Mut% in duplicate. Exceptions are the 20 %, 50 %, and 80 % levels, which were tested in triplicate (for raw data of 3 precision runs [run#1–3], see Supplement tab gBlock samples). We achieved a linear response with a slope of 1.06, and R^2^ = 0.995 (by plotting the E_295_K Mut% results from the AS LNA qPCR against the E_295_K Mut% input quantity of the synthetic dsDNA fragments, Fig. 2A). Probe specificity was confirmed using the 0 % and 100 % E_295_K Mut% levels (corresponding to 100 % WT and 100 % Mut gBlock, respectively), resulting in no detection of the alternate probe.

Additional probe specificity was confirmed for two known biologically relevant mutations occurring in the probe region, using 100 % gBlock-E_295_G or 100 % gBlock- P_294_S input, which resulted in negligible detection when using the E_295_K-WT or Mut probes for detection (Fig. 2B). To judge the relevance of the co-mutation for manufacturing processes, the Integrative Genomics Viewer (IGV) v2.5.3 (https://igv.org/) was used to check for the existence of these mutations on the same reads or if they always occurred on separate reads (of the short read NGS library). Indeed, E_295_K-Mut E_295_G and P_294_S never co-existed on the same reads, therefore it was concluded that the biological population contains only viruses with one of these 3 mutations, and never a combination. Variation within runs did not exceed 2 % RSD, illustrating high precision. Also, between runs, the variation was on average 2.8 % RSD (Fig. 2C,D). The variation of the RT reaction was not included in this comparison, since synthetic dsDNA fragments were used for these determinations. Next, we extended the reproducibility testing to sPV2 poliovirus samples (Fig. 2E). Here, 16 samples with increasing E_295_K Mut% were tested in two independent assay runs. The average difference between the sample results between the runs was 1.2 % and the maximum difference was 4.9 %.

### 3.4 High correlation between qPCR and NGS results in Sabin poliovirus type 2 samples

**Fig. 3:**
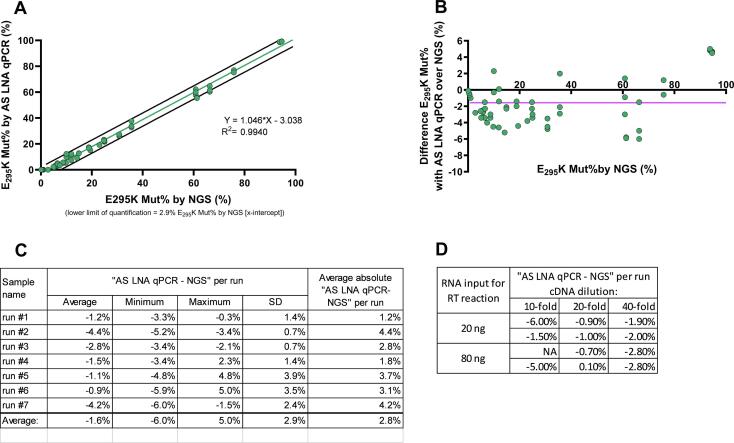
Qpcr accuracy on sabin poliovirus type 2 samples compared to ngs. (A) Correlation between E_295_K Mut% measured by NGS and by the established AS LNA qPCR from *n* = 58 sPV2 samples, tested in 8 independent qPCR runs, including the best-fit linear regression line in green and the 95 % prediction bands in black. (B) Differences between E_295_K Mut% detected in NGS and AS LNA qPCR per sample tested. The purple line shows the average difference between the two methods (-1.6 %). (C) Differences between E_295_K Mut% in NGS and AS LNA qPCR averaged per run. (D) Impact of cDNA matrix on AS LNA qPCR accuracy (*n* = 1 sPV2 sample, tested 12x; NA = invalid measurement). (For interpretation of the references to colour in this figure legend, the reader is referred to the web version of this article.)

With a linearity of R^2^ > 0.994, the expected percentages of E_295_K mutants and those determined by NGS correlated well with the percentages determined with our probe-based AS LNA qPCR method. The lower quantification limit was 2.9 % (95 %CI 2.032–3.754) E_295_K Mut% by NGS (X-intercept). Considering the 95 % confidence interval, the limit is thus 3.75 %. At NGS levels of E_295_K of ≥3.75 %, our AS LNA qPCR can thus reliably detect the E_295_K mutation (Fig. 3A,B).

Quantification of the difference between NGS and qPCR results showed that on average the mutational load was underestimated by qPCR by 1.6 % compared to NGS (*n* = 58). The lowest underestimation reached 6.0 % and the overestimation 5.0 % E_295_K. The absolute difference between AS LNA qPCR and NGS results was 2.8 % (Fig. 3C). On average, the mutational load was underestimated by qPCR by 2.3 % for samples with an NGS mutational percentage of <40 %, while the samples with a higher E_295_K mutational load (≥40 %) were only underestimated by 0.2 % in the qPCR, compared to NGS. However, the variation in the data obtained for samples with higher E_295_K mutational load was higher (SD of 4.3 % for ≥40 % E_295_K Mut% versus SD of 1.7 % for <40 % E_295_K Mut%) and the sample size was lower (*n* = 16 for ≥40 % E_295_K versus *n* = 42 for <40 % E_295_K Mut%, see raw data Supplement, tab accuracy analysis).

By applying a 2-fold or 4-fold higher dilution of the input cDNA, compared to the standard dilution of 10-fold, applied for the data collection, as summarized in the results above, the underestimation of the qPCR method was improved from 4.2 % to 0.6 % or 2.4 %, respectively (Fig. 3D). For this test, one sPV2 sample (66.3 % E_295_K in NGS), was tested 4 times at each of the targeted cDNA dilutions. Moreover, the results obtained with the 20-fold or 40-fold diluted cDNA showed more stable fluorescence baselines.

## 4 Discussion

Poliovirus vaccine manufacturing and batch release need to be closely monitored regarding the occurrence of undesired mutations affecting neurovirulence or immunogenicity. Here, we present the first AS LNA qPCR method for the targeted, specific, and high-throughput detection of the frequently occurring, and potentially immunogenicity-reducing, mutation E_295_K in poliovirus vaccines, or more specifically type 2 serotype of sIPV. Our data shows that our developed AS LNA qPCR is highly reproducible and specific for the E_295_K mutation, renders comparable results to NGS analysis per studied sample, and could thus be used as low-cost and quick pre-screening technology before or instead of NGS.

The majority of mutation-bearing sPV2 batches for sIPV manufacturing contain the E_295_K mutation [24], [25]. The impact of this mutation on systemic and mucosal antigenicity/immunogenicity has not been reported yet for intramuscular vaccination. But, in the case of nOPV2 vaccines, the E_295_K mutation causes loss of antigenicity/immunogenicity [16]. The E_295_K mutation is currently biologically best characterized for nOPV2 vaccines [22], [26] and thus closely monitored during manufacturing processes using costly and time-consuming NGS analysis. The here-developed AS LNA qPCR assay correlates well with NGS data regarding E_295_K detection and quantification and may present a low-cost, high-throughput pre-screening alternative to NGS. In particular for the nOPV2 candidate 1, which contains a Sabin-2 capsid [10], our AS LNA qPCR method may be directly applicable. However, for nOPV2, the co-location of E_295_K and N_171_D in VP1 is common and biologically consequential [27], and here long-read sequencing may be required to measure this co-location. The here presented AS LNA qPCR assay demonstrated high sensitivity by accurately identifying mutation-positive samples that surpassed the established detection limit. However, the AS LNA qPCR slightly underestimated mutation-positive results in comparison to NGS, where all samples tested mutation-positive. The determination of the detection limit, set at 3.75 % E_295_K Mut% (as determined by NGS), was based on the X-intercept (including 95 % confidence interval) of the correlation between NGS and AS LNA qPCR (Fig. 3A). This data points towards a less optimal probe-binding in case of low mutation abundance in the sample, as inherent to the qPCR technology (LOQ, LOD). Thresholds regarding proceeding with manufacturing for low-Mut% samples will thus need to be redefined for our technology, after additional attempts to enhance the sensitivity. Based on our current analysis, we suggest reliable use of the AS LNA qPCR for E_295_K mutation detection down to a detection limit of 3.75 %. Samples with AS LNA qPCR-based Mut%- below that threshold should be re-assessed using NGS, before clearing them for manufacturing.

The underestimation of the qPCR method, compared to NGS, may be partially caused by the impact of the cDNA matrix (containing DTT). We found that a minimum 20-fold dilution of the cDNA matrix in distilled water was required to stabilize the fluorescent signal of the qPCR, leading to more robust and more accurate results, while most of our experiments were conducted using a suboptimal 10-fold diluted cDNA matrix. Efficiency differences between the two probes could additionally explain the found inaccuracies of the qPCR method. These efficiency differences could lead to inaccuracies in the proportional quantification of WT versus Mut. Here, efficiency correction, as reported by Ruijter et al. [28], could be an effective approach for the accurate calculation of the SNP frequency, by compensating for the PCR efficiency differences. Yet, probes remain highly specific and off-target binding of the LNA probes, despite not tested, is not expected based on our experience and the enhanced affinity and mismatch discrimination properties of the probes. Translating this finding to manufacturing and screening praxis, E_295_K-mutation-negative results from our technology do not exclude the presence of other co-mutations. Yet, it may reduce the number of samples to be screened by NGS. Therefore, the AS LNA qPCR presented here is estimated to save tremendous NGS costs and time. From the target-specificity of our probes, however, evolves the need for the integration of additional SNPs in the assay for broader screening of potentially arising biologically relevant SNPs if desired. Here, this AS LNA qPCR is highly suitable for broadening its applicability and adding additional SNPs and targets to the qPCR, due to the characteristics of the LNA probes (increased affinity, increased mismatch discrimination, and the full control of melting point of the hybridization reaction [29]). Indeed, from these characteristics, LNA-modified nucleotides have been used for a variety of applications, such as *in situ* hybridization [30], whole genome amplification [31], germline SNP genotyping [32], [33], viral differentiation SARS-CoV-2 from influenza A and B [34], as blocker LNA oligonucleotide for the detection of oncogene mutations with high sensitivity, including *KRAS* and *BRAF* [35], and recently as allele-specific primers for accurate and cost-effective diagnosis and quantification of *KRAS* and *BRAF* mutations [36]. Here, we are the first to apply this technology for vaccine development screening purposes or vaccine batch release.

Similarly, from these characteristics, the AS LNA qPCR is likely easily extended to include neurovirulence-inducing mutation probes, such as 5′UTR nucleotide 481-G, VP1-I_143_T, or VP1-N_171_D (frequently occurring and biologically impacting in nOPV2 [27]) for screening of sPV2 and/or nOPV2 batches. A limitation of the qPCR method is that it will not be suitable for detecting co-location of different variants in a single genome. Depending on the position of the mutations of interest, either short-read or long-read sequencing will be needed to determine if mutations are co-locating. Manukyan et al. recently published a quantitative multiplex, one-step RT-PCR for assessing selected neurovirulence mutations and the E_295_K mutation [22]. Though a direct comparison between accuracy, specificity, and sensitivity results is not feasible due to the different materials used, our AS LNA-based technology, has major advantages over the Manukyan-approach: The presented LNA incorporation in the probes leads to higher annealing temperatures (Tms) of probes, allowing for shorter probe lengths with better quenching and a higher signal-to-noise ratio and thus an improved ability to distinguish SNPs. Additionally, we present a two-step approach. Though more time-consuming than a one-step approach, the sensitivity is often better in a two-step methodology (as both steps of the process can be optimized), which is an absolute necessity in application-relevant screening technology.

Importantly, NGS has the advantage over our AS LNA-based technology of the direct enrichment of the target sequence in the coverage of highly mutable viruses even at low concentrations in the pathogen samples. However, in cases where sequences are known or sequence variability is low, the AS LNA qPCR may replace NGS and be more cost-effective. To avoid possible limitations of LNA probes (e.g., in case of degenerate sequences of pathogens), and to increase targeted enrichment, overlapping primers/LNA probes containing degenerate sequences can be designed and applied, as previously successfully demonstrated [37]. In addition, an increased sensitivity of LNA binding to the target can be obtained by adding LNA blockers [38] or by using LNA probes in combination with ultra-degenerate primers with 3′ termini overlapping the probe-binding site (Pan-Degenerate Amplification and Adaptation (PANDAA)) [39].

## 5 Conclusions

The developed AS LNA qPCR for E_295_K mutations is highly sensitive for detecting mutation-positive samples and can thus be useful as a cost-effective and quick pre-screening tool for poliovirus vaccines.

## 6 Financial support

Financial support for this work was received from the 10.13039/100000865Bill & Melinda Gates Foundation (BMGF, grant numbers: INV-007268 (formerly: OPP1203429), OPP1210558, and INV-030972), which did not influence the outcomes of the research. All authors confirm to have contributed to the manuscript, and that the manuscript has been read and approved for submission by all the named authors.

## 7 Transfer of copyrights

The copyright of the manuscript will not be transferred to the journal publisher upon acceptance for publication. This work was supported, in whole or in part, by the Bill & Melinda Gates Foundation Grant Numbers: INV-007268 (formerly: OPP1203429), OPP1210558, and INV-030972. Under the grant conditions of the Foundation, a Creative Commons Attribution 4.0 Generic License has already been assigned to the Author Accepted Manuscript version that might arise from this submission.

## CRediT authorship contribution statement

**Lizet Opmeer:** Investigation, Data curation, Formal Analysis. **Isabella Gazzoli:** Conceptualization, Investigation, Data curation, Formal Analysis. **Monika Ballmann:** Investigation, Methodology, Formal Analysis. **Marieke Willemsen:** Project administration, Resources. **Gerben Voshol:** Investigation, Data curation, Formal Analysis. **Magda Grudniewska-Lawton:** Investigation, Data curation, Formal Analysis. **Menzo Havenga:** Visualization, Writing – review & editing. **Christopher Yallop:** Funding acquisition, Conceptualization, Supervision. **Ahd Hamidi:** Funding acquisition, Conceptualization, Supervision. **Gert Gillissen:** Investigation, Methodology, Formal Analysis. **Wilfried Bakker:** Visualization, Writing – original draft, Writing – review & editing.

## Declaration of competing interest

The authors declare that they have no known competing financial interests or personal relationships that could have appeared to influence the work reported in this paper.

## Data Availability

The authors confirm that the data supporting the findings of this study are available within the article and/or its supplementary materials.

## Appendix A Supplementary material

Raw data, including protocol optimization, standard curves, and linearity, precision, and accuracy assessment data, supporting the findings and discussions presented in this paper.

Supplementary data 1 Supplementary equations: standard equations use.

Supplementary data 2 Raw data Excel file 1: raw linearity data using gBlock E295K-WT and E295K-Mut standard curves as input, including Mut% input and measured Mut%, calculated Mut% difference, and absolute Mut% difference, and raw data NGS vs. AS LNA qPCR E295K mutation detection in sPV2 samples.
