# Supplementary material for: High throughput AS LNA qPCR method for the detection of a specific mutation in poliovirus vaccine strains
Source: Vaccine. 2024 Apr 2;42(9):2475–84. doi: 10.1016/j.vaccine.2024.01.103 (PMC11007389; doi:10.1016/j.vaccine.2024.01.103)
Supplement: Supplementary data 1 — Supplementary equations: standard equations use. [file mmc1.docx]

| **Equation S1: Conversion from nanograms to copy numbers** |
| --- |
| $Copy number \left[ copies/\mu L \right]=\frac{\left( \frac{Weight \left[ g \right]}{Molecular weight \left[ g/\mathrm{mole} \right]} \times6.022 x 10^23 \left[ \mathrm{copies}/\mathrm{mole} \right] \right)}{Volume \left[ \mu L \right]}$ |

| **Equation S2: Quantities calculation, using standard curve linear regression line parameters** |
| --- |
| $Quantity \left[ \mathrm{copies}/{\mu L} \right]= {10}^{\frac{Ct-Intercept}{Slope}}$ |

| **Equation S3: PCR efficiency of the standard curve** |
| --- |
| $Efficiency \left[ \% \right]= \left( {10}^{\frac{-1}{Slope}}-1 \right) x 100\%$ |
